# High-Grade Chondrosarcoma Involving the Xiphoid Process and Lower Sternum—The Continuing Role of Extended Open Chest Wall Surgery in the Era of Minimally Invasive Thoracic Surgery: A Case Report

**DOI:** 10.3390/curroncol33090544

**Published:** 2026-09-09

**Authors:** Thomas Rallis, Maria Mironidou-Tzouveleki, Vasileios Theocharidis, Apostolos Gogakos, Dimitrios Paliouras, Achilleas Lazopoulos, Nikolaos Christoglou, Panagiotis Panousis, Michael Katsamakas, Paraskevas Vrochidis, Meropi Koutourini, Myrto Tzinevi, Pipitsa Valsamaki, Alexandra Mpakosi, Nikolaos Barmpetakis

**Affiliations:** 1 Thoracic Surgery Department, “Theageneio” Cancer Hospital, 54639 Thessaloniki, Greece; theocharidisvassilis@gmail.com (V.T.); drgogakos@hotmail.com (A.G.); demtros@yahoo.gr (D.P.); axlazopoulos@hotmail.com (A.L.); nikolaos.christoglou@gmail.com (N.C.); mkoutourini@hotmail.com (M.K.); myrtwtzinevi@gmail.com (M.T.); nibarbet@yahoo.gr (N.B.); 2 First Laboratory of Pharmacology, Medical School, “Aristotle” University of Thessaloniki, 54124 Thessaloniki, Greece; mmyronidauth@gmail.com; 3 Surgical Oncology Department, “Theageneio” Cancer Hospital, 54639 Thessaloniki, Greece; panagiotis.panousis@gmail.com (P.P.); makat@med.auth.gr (M.K.); 4 ENT Department, General Hospital of Serres, 62100 Serres, Greece; paref25@gmail.com; 5 PET Unit, Nuclear Medicine Department, Medical School, Democritus University of Thrace, 69100 Panepistimioupoli, Greece; pivalsam@med.duth.gr; 6 Department of Immunology, General Hospital of Nikaia “Agios Panteleimon”, 18454 Piraeus, Greece; alexiabakossi@yahoo.gr

**Keywords:** chondrosarcoma, clamshell, thoracic, surgery

## Abstract

Primary chondrosarcoma of the sternum is a rare malignancy whose anatomical location can create major diagnostic, resection, and reconstruction challenges. We report a 62-year-old man with severe obesity and a heavy smoking history who presented with a progressively enlarging, painful sternal swelling. Preoperative imaging conducted via computed tomography and positron emission tomography–computed tomography demonstrated a large heterogeneous mass at the anterior thoracoabdominal junction infiltrating the xiphoid process. CT-guided biopsy established grade III chondrosarcoma. Because of the tumor’s size, location, and infiltrative features, bilateral transverse thoraco-sternotomy (“Clamshell” Procedure) was performed. “En bloc” resection included the middle and lower sternum, involved soft tissues and adjacent costal arches, followed by prosthetic synthetic polypropylene mesh reconstruction. The final pathology documented tumor-free examined margins (R_0_), with an approximately 10 mm closest reported clearance. The postoperative course was prolonged and complex. The patient required 18 days of intensive care and tracheostomy on postoperative day 8 because of delayed awakening and intermittent oxygen desaturation. Wound dehiscence necessitated surgical revisions on postoperative days 29 and 44; during the second revision, the infected mesh was removed. The tracheostomy was removed on day 64, and the patient was discharged in a stable condition two days later. This case demonstrates that extensive open approaches remain indispensable for selected chest wall malignancies. It also emphasizes the importance of coordinated multidisciplinary perioperative care from diagnosis through recovery, while illustrating how reconstructive resources, patient risk, and infection can determine the procedure’s outcome.

## Simple Summary

Chondrosarcoma is a rare cancer of cartilage that can affect the breastbone. Large tumors in this area are difficult to remove due to the proximity to important structures, eventually leaving a remaining defect in the chest wall. This report describes a 62-year-old man with a large, aggressive tumor on the lower breastbone. The tumor was removed through an extensive open operation with additional application of surgical synthetic polypropylene mesh. Recovery was prolonged and complicated by breathing difficulties and wound infection, requiring further operations and removal of the mesh. The patient was eventually discharged in a stable condition. This case shows that traditional open surgery remains essential for some complex chest wall cancers, despite advances in minimally invasive surgery. It also highlights the need for coordinated care by several medical specialties. Future studies should identify safer reconstruction methods, reduce postoperative complications, and support referral pathways to experienced specialist centers, combining thoracic, sarcoma, and reconstructive expertise.

## 1. Introduction

Chondrosarcoma is a malignant bone tumor characterized by the production of cartilaginous matrix by neoplastic cells. Although it represents one of the most frequent primary malignant bone tumors in adults, its occurrence in the thoracic cage remains relatively uncommon when compared with more typical axial or appendicular skeletal locations [1]. Within the chest wall, chondrosarcoma most frequently arises from the ribs, costochondral junctions, or sternum. Tumors involving the lower sternum and xiphoid process are particularly rare, and their anatomical position at the transition between the thoracic and upper abdominal wall creates diagnostic, surgical, and reconstructive difficulties that differ from those encountered in more lateral costal lesions [2].

Chest wall chondrosarcomas may present as slowly enlarging masses, frequently accompanied by pain, local tenderness, respiratory discomfort, or deformity of the anterior thoracic contour. Their clinical course can be deceptively indolent, especially in low-grade lesions. However, high-grade tumors, including grade III chondrosarcomas, carry substantially more aggressive biological potential, with a greater risk of local recurrence, metastatic dissemination, and disease-related mortality. The histological grade, anatomical site, tumor size, adequacy of surgical margins, and feasibility of complete resection are therefore central determinants of prognosis [3]. Therefore, complete “en bloc” resection with negative margins remains the principal curative treatment [4]. Contemporary national data confirm that positive margins are associated with substantially worse survival, while recent multicenter data suggest that a microscopically negative clearance of at least 4 mm improves local control [5]. The operation must therefore balance oncologic clearance against the respiratory and mechanical consequences of the resulting defect [6].

The diagnostic workup of suspected chest wall chondrosarcoma requires close integration of clinical assessment, cross-sectional imaging, nuclear medicine when appropriate, and histopathological confirmation. Computed tomography (CT) is particularly useful for defining cortical destruction, mineralized chondroid matrix, calcifications, and the relationship of the tumor to the ribs, sternum, diaphragm, mediastinum, and upper abdominal structures. Magnetic resonance imaging (MRI) provides complementary information regarding soft-tissue extension, marrow involvement, neurovascular proximity, and the extent of local invasion into adjacent muscular or fascial planes. Positron emission tomography–computed tomography (PET–CT) contributes to staging, metabolic characterization, detection of distant disease, and postoperative surveillance, although its findings must always be interpreted in relation to anatomical imaging and tissue diagnosis. Three-dimensional segmentation may improve margin and implant planning in selected complex defects, but it requires dedicated technical infrastructure and funding.

From a therapeutic perspective, conventional chondrosarcoma remains primarily a surgical disease. Unlike osteosarcoma or Ewing sarcoma, conventional chondrosarcoma is generally considered relatively resistant to standard chemotherapy and radiotherapy. Consequently, wide “en bloc” surgical excision with tumor-free margins remains the cornerstone of curative-intent management. Radiotherapy may have a role in unresectable disease, close or positive margins, palliation, or selected anatomical sites where complete surgical clearance is not feasible. Systemic therapy is usually limited, although selected subtypes, such as mesenchymal or dedifferentiated chondrosarcoma, may justify individualized oncologic treatment strategies [7].

Modern thoracic surgery has been transformed by minimally invasive techniques, including video-assisted (VATS) and robotic-assisted thoracoscopic surgery (RATS). These approaches have improved postoperative recovery, reduced surgical trauma, and become standard practice in many intrathoracic oncologic and non-oncologic diseases. Nevertheless, large anterior chest wall tumors with sternal invasion, extensive bony involvement, or a requirement for complex reconstruction often remain outside the safe limits of minimally invasive surgery. In such circumstances, traditional open surgical approaches retain a decisive role, not as outdated techniques, but as essential components of contemporary oncologic thoracic surgery.

The “Clamshell” procedure, also known as bilateral transverse thoraco-sternotomy, although less frequently used in routine elective thoracic surgery, offers wide bilateral exposure of the anterior thoracic cavity, sternum, mediastinum, pleural spaces, and cardiopulmonary structures. In selected cases, this access may be indispensable for safe tumor mobilization, vascular control, bilateral exploration, and immediate reconstruction of large anterior defects [8]. Large anterior or central defects may require flexible mesh, rigid plating, methyl methacrylate, custom implants, vascularized soft-tissue flaps, or a hybrid construct. No single method is universally superior, and selection depends on defect geometry, contamination risk, available tissues, institutional expertise, and cost. This case report illustrates the importance of individualized operative planning in a patient with high-grade xiphoid/lower sternal chondrosarcoma, while focusing on the operative reasoning, limitations, and complications of a “Clamshell” resection and mesh-only reconstruction. It also highlights the postoperative complexity of such surgery, including intensive care management, respiratory support, tracheostomy, wound dehiscence, prosthetic mesh infection, surgical revisions, and the need for prolonged multidisciplinary follow-up [9].

## 2. Clinical Presentation

**Figure 1:**
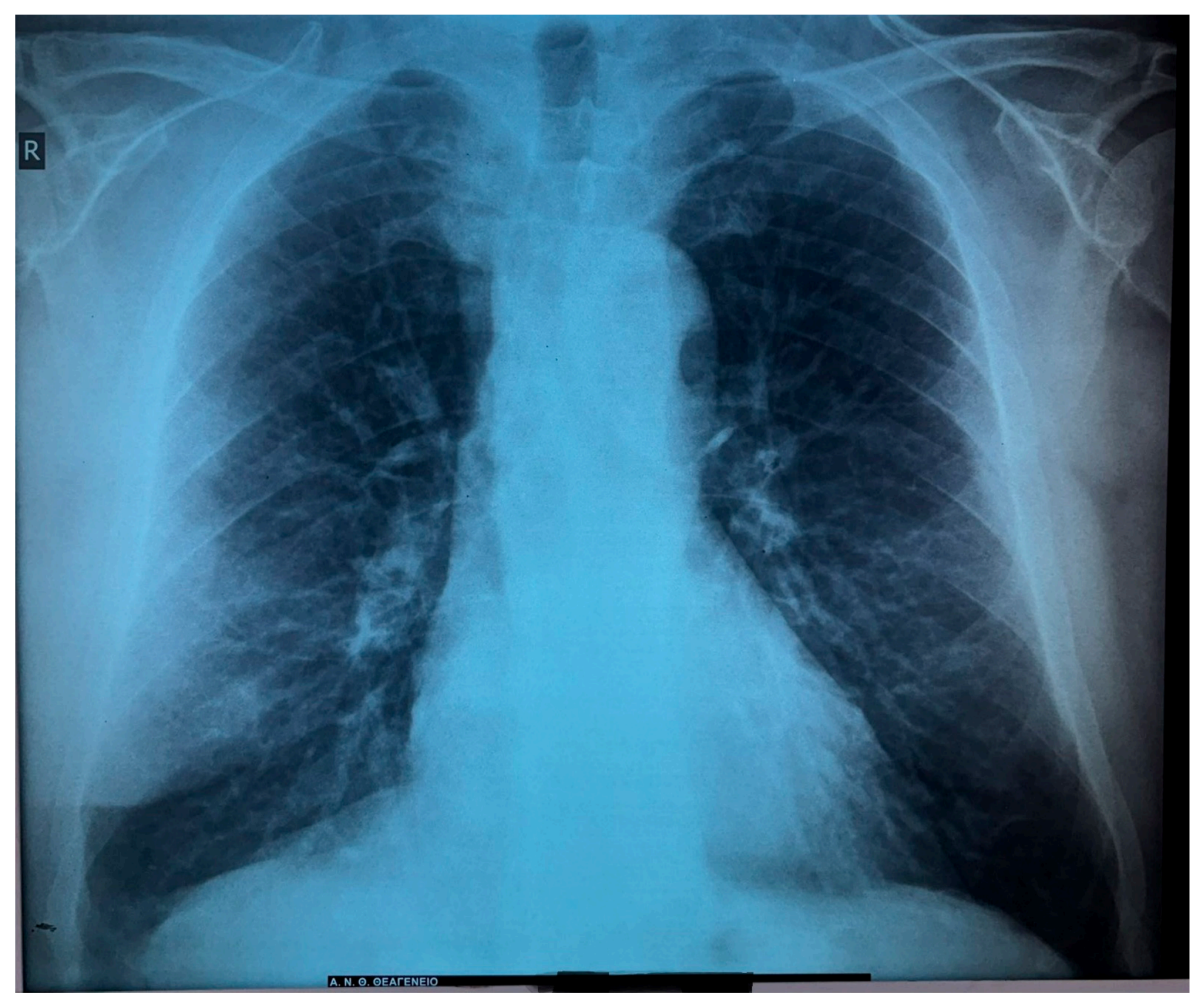
Preoperative X-ray control.

A 62-year-old male patient was referred for evaluation of a progressively enlarging and increasingly painful swelling in the lower sternal region. The patient had no previous oncologic history. His risk medical profile was notable for obesity, with a body weight of 127 kg and a high body mass index (BMI), as well as a history of heavy smoking (Figure 1). These clinical characteristics were relevant not only to perioperative risk assessment, but also to the postoperative ventilation, respiratory recovery and wound-healing capacity.

**Figure 2:**
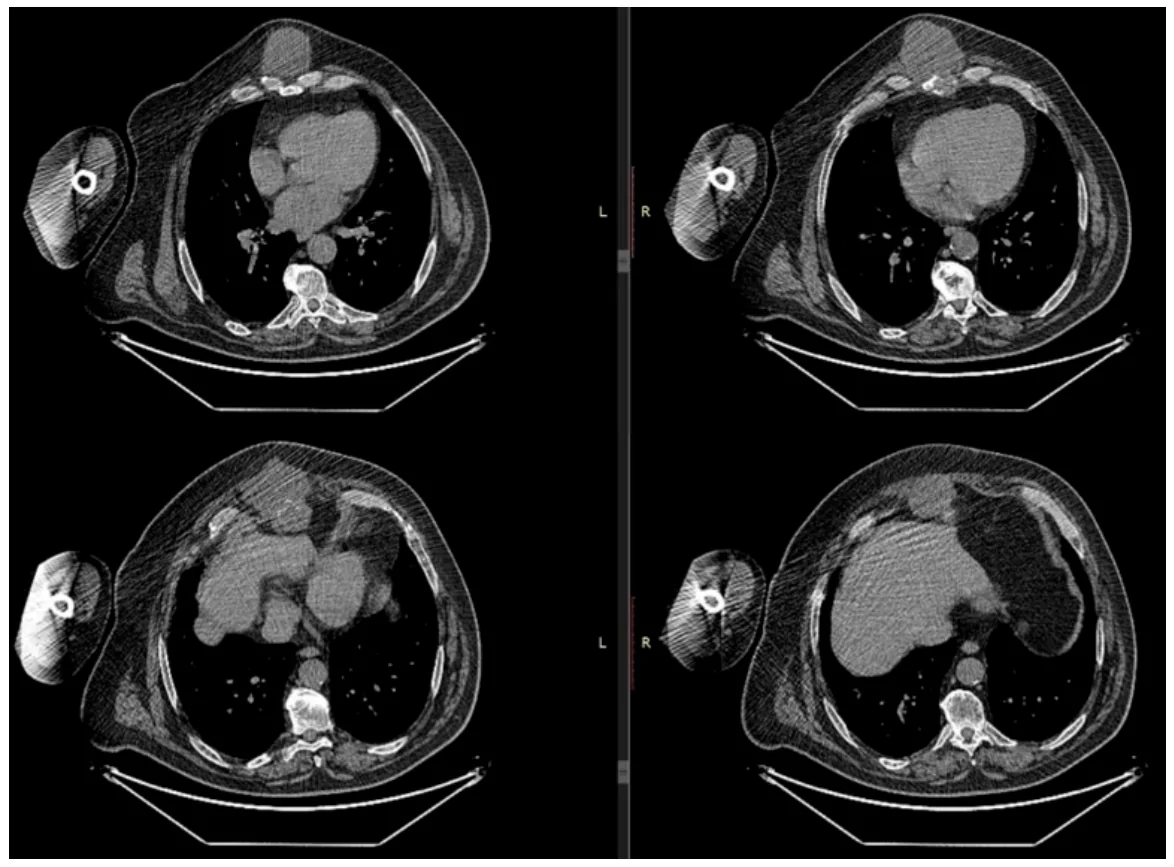
Preoperative axial CT images demonstrating the lower anterior chest wall lesion and its relationship to the thoracoabdominal junction.

**Figure 3:**
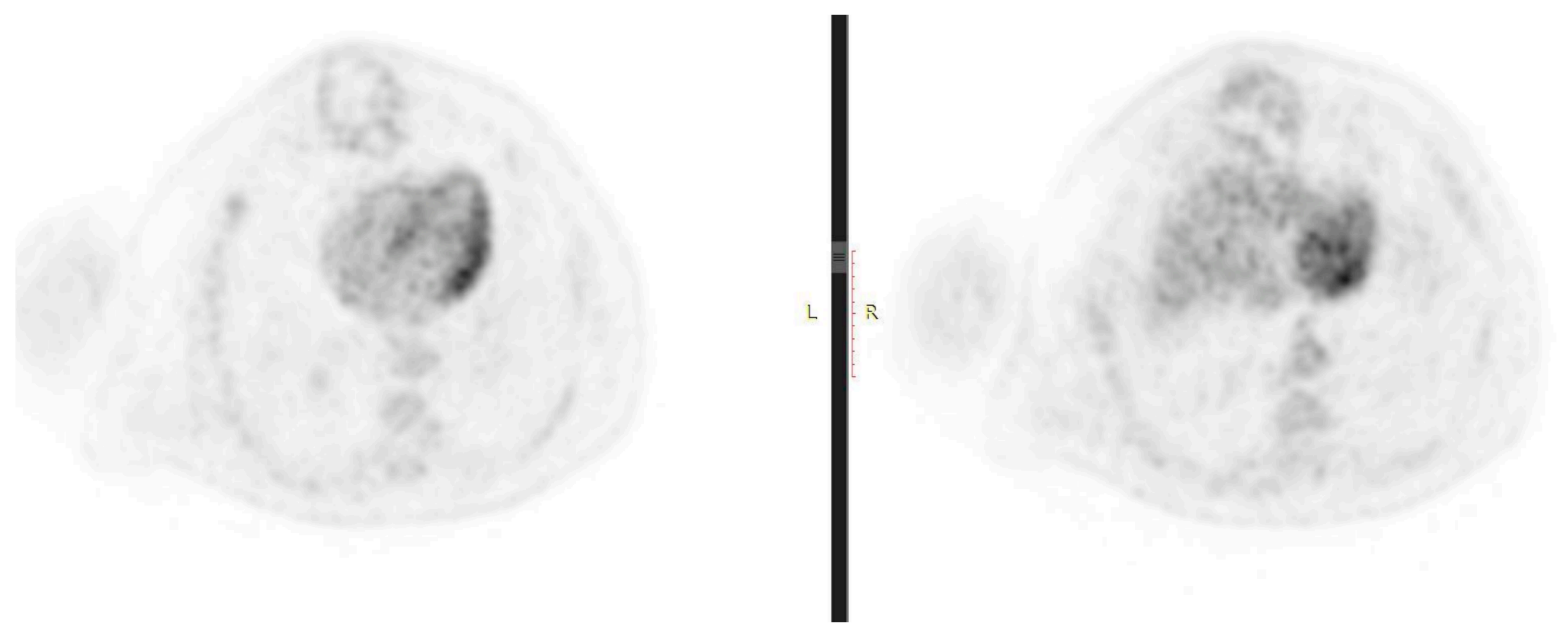
Preoperative axial PET–CT staging captions in inverted grayscale.

**Figure 4:**
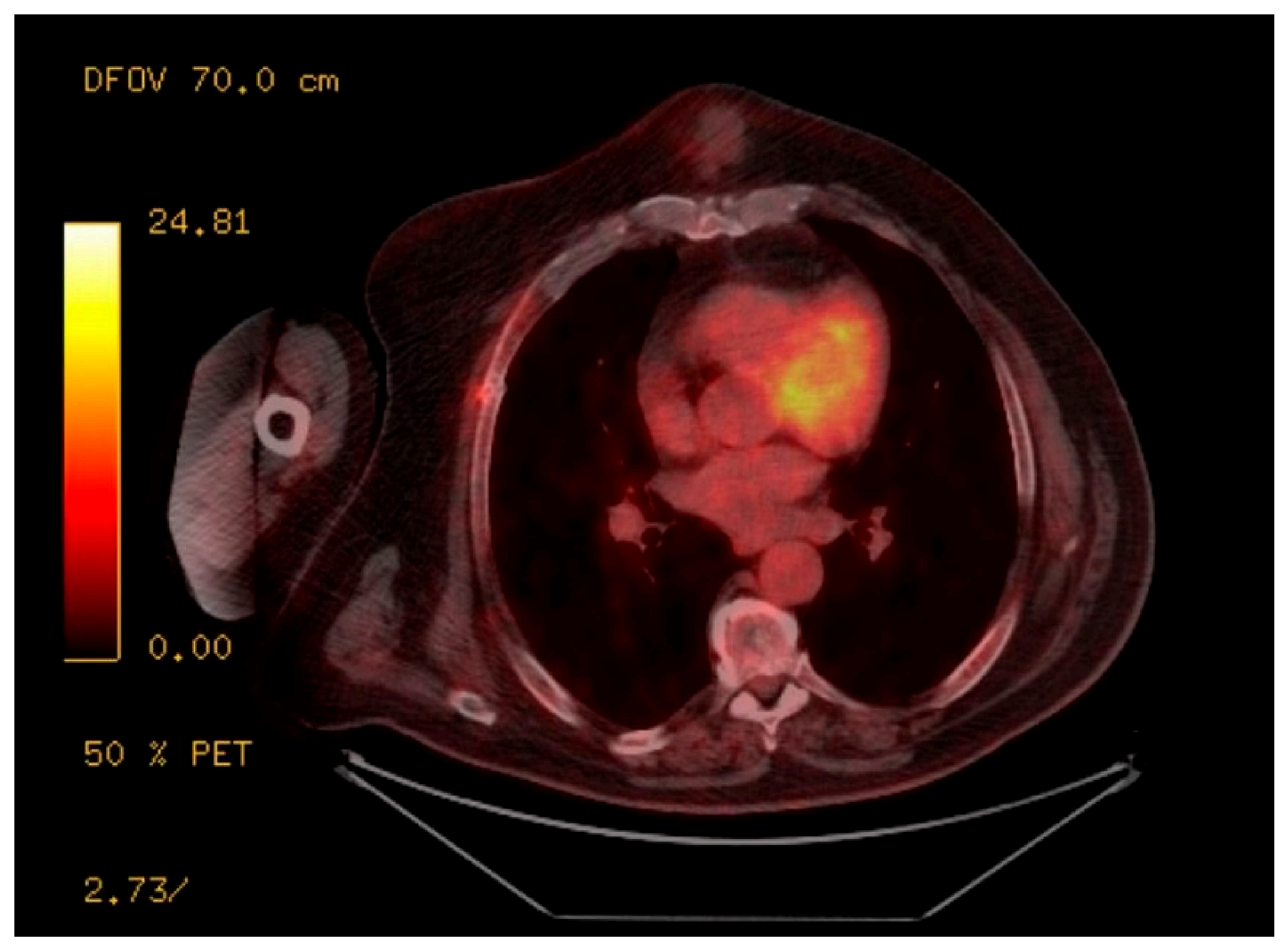
Preoperative axial fused PET/CT caption through the lower thorax at the level of the heart.

**Figure 5:**
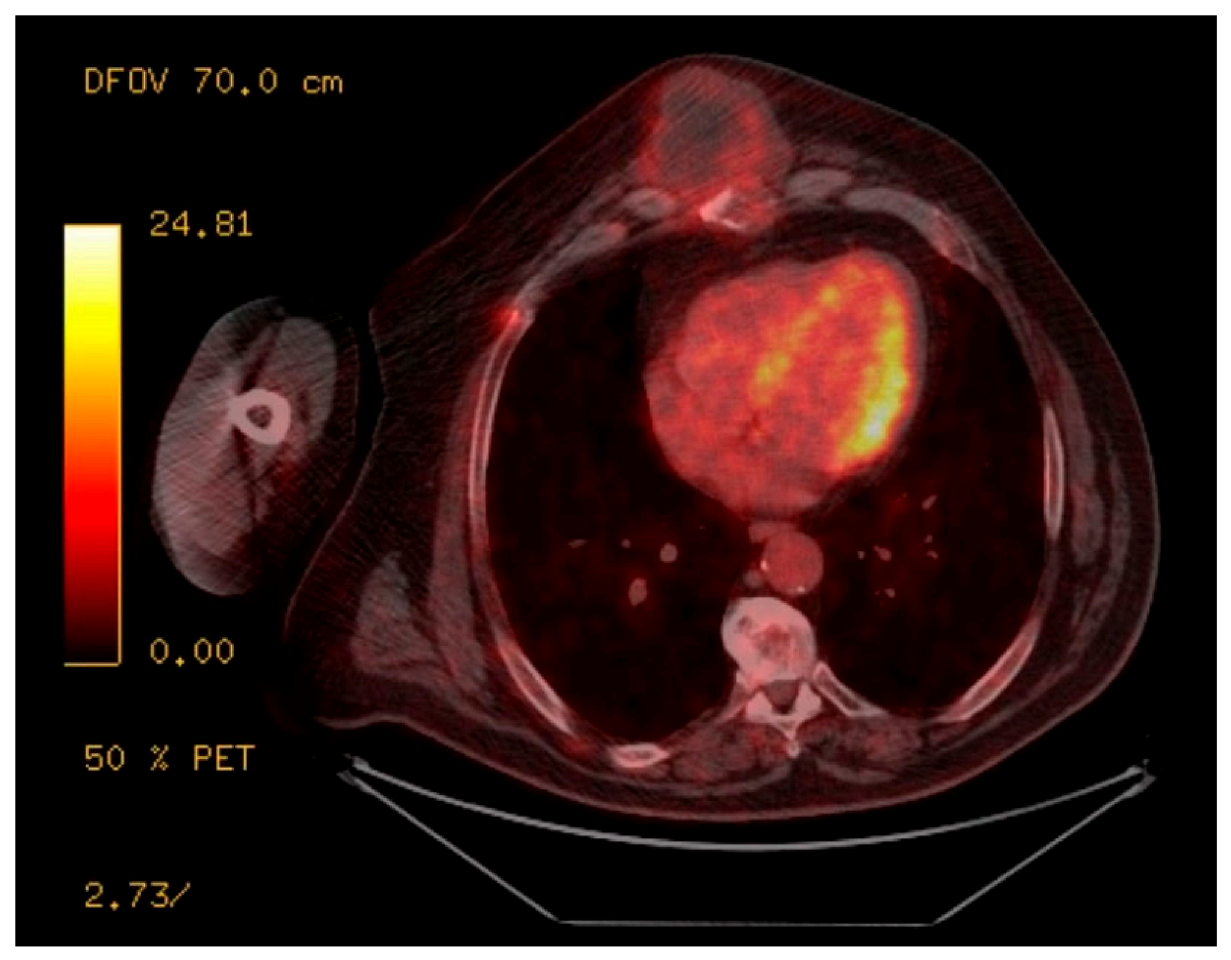
Preoperative axial fused PET/CT caption at the level of the lower heart and lower sternum.

**Figure 6:**
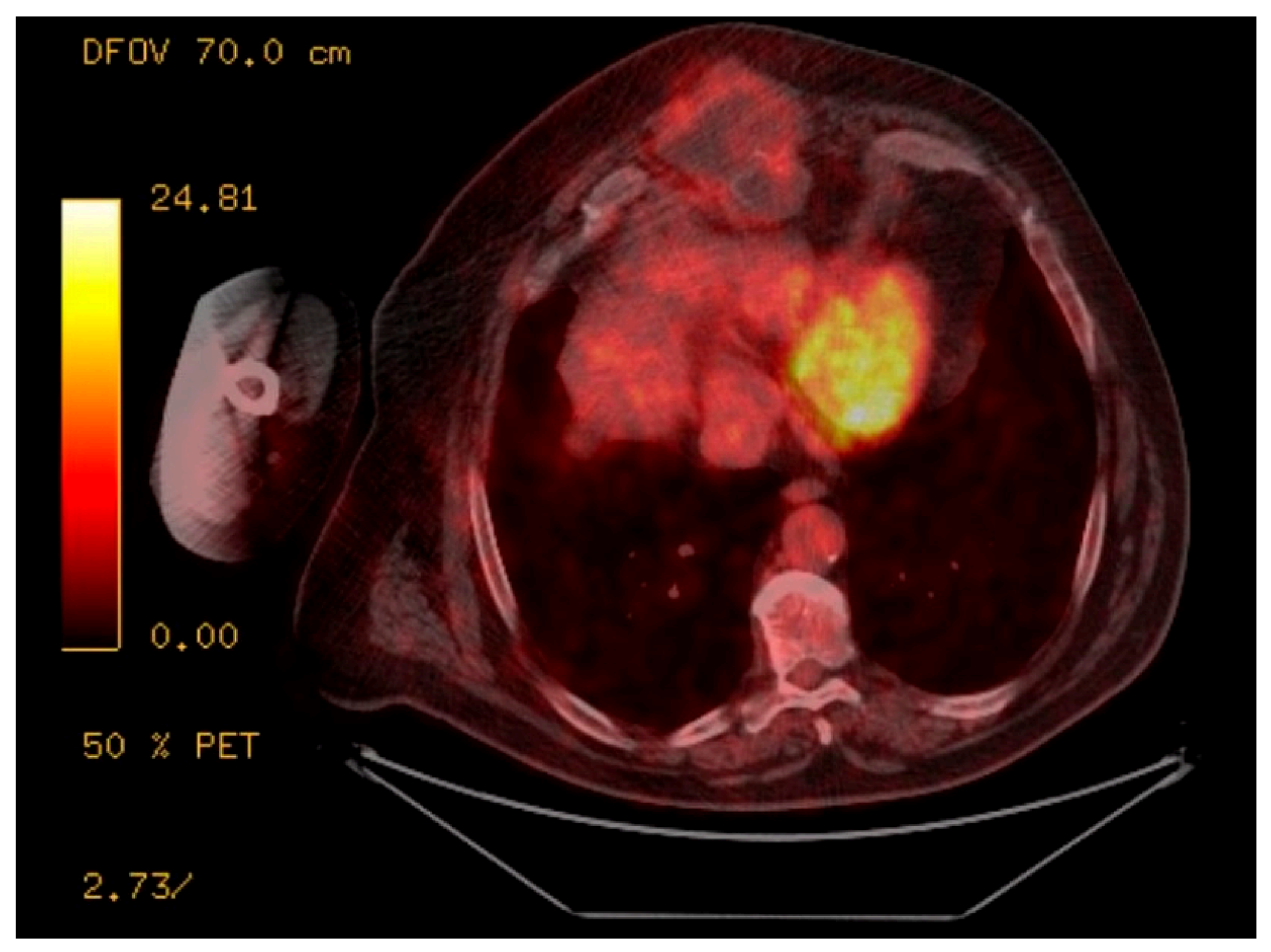
Preoperative axial fused PET/CT caption through the inferior thorax, near the cardiophrenic and diaphragmatic levels.

**Figure 7:**
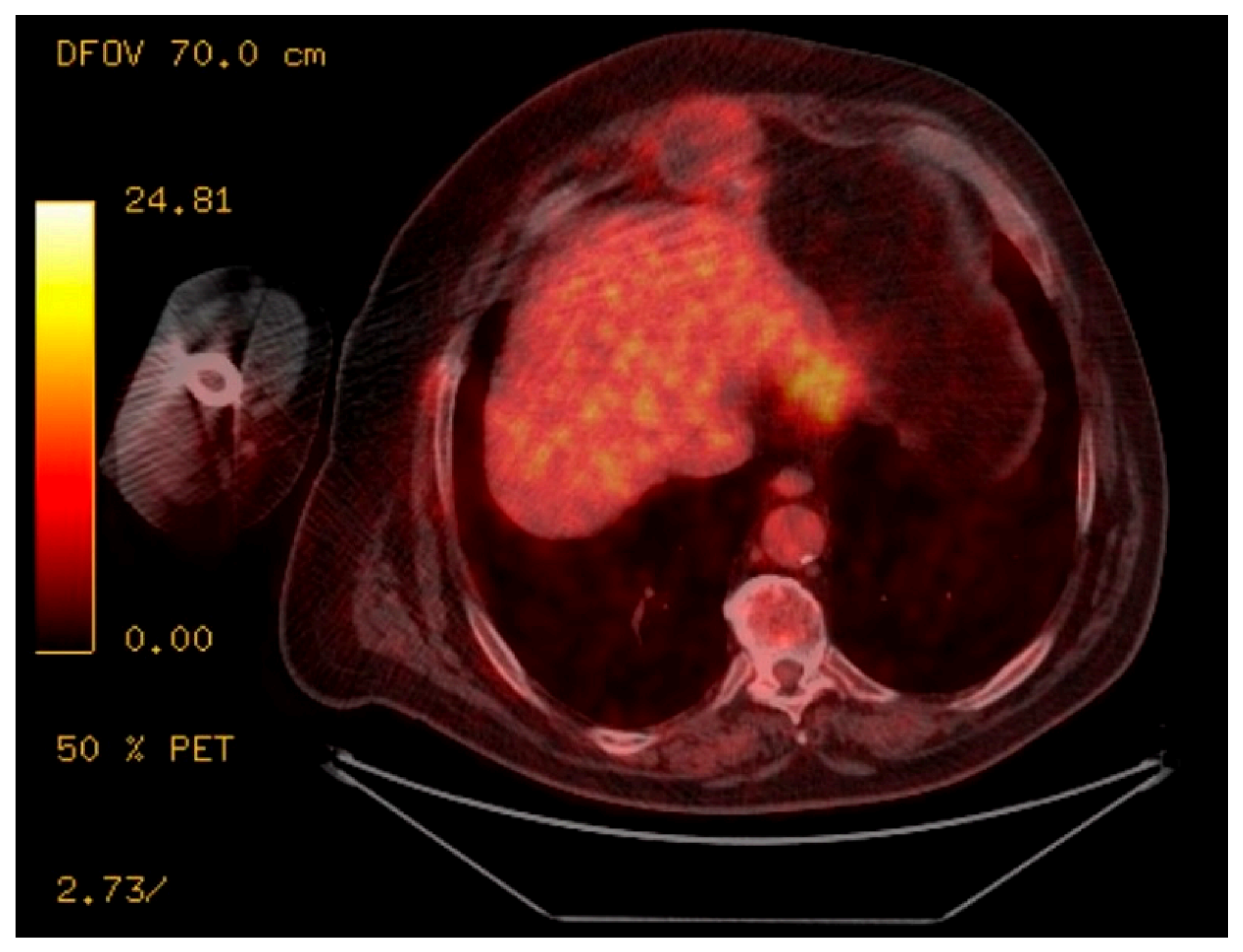
Preoperative axial fused PET/CT caption at the level of the thoracoabdominal junction.

**Figure 8:**
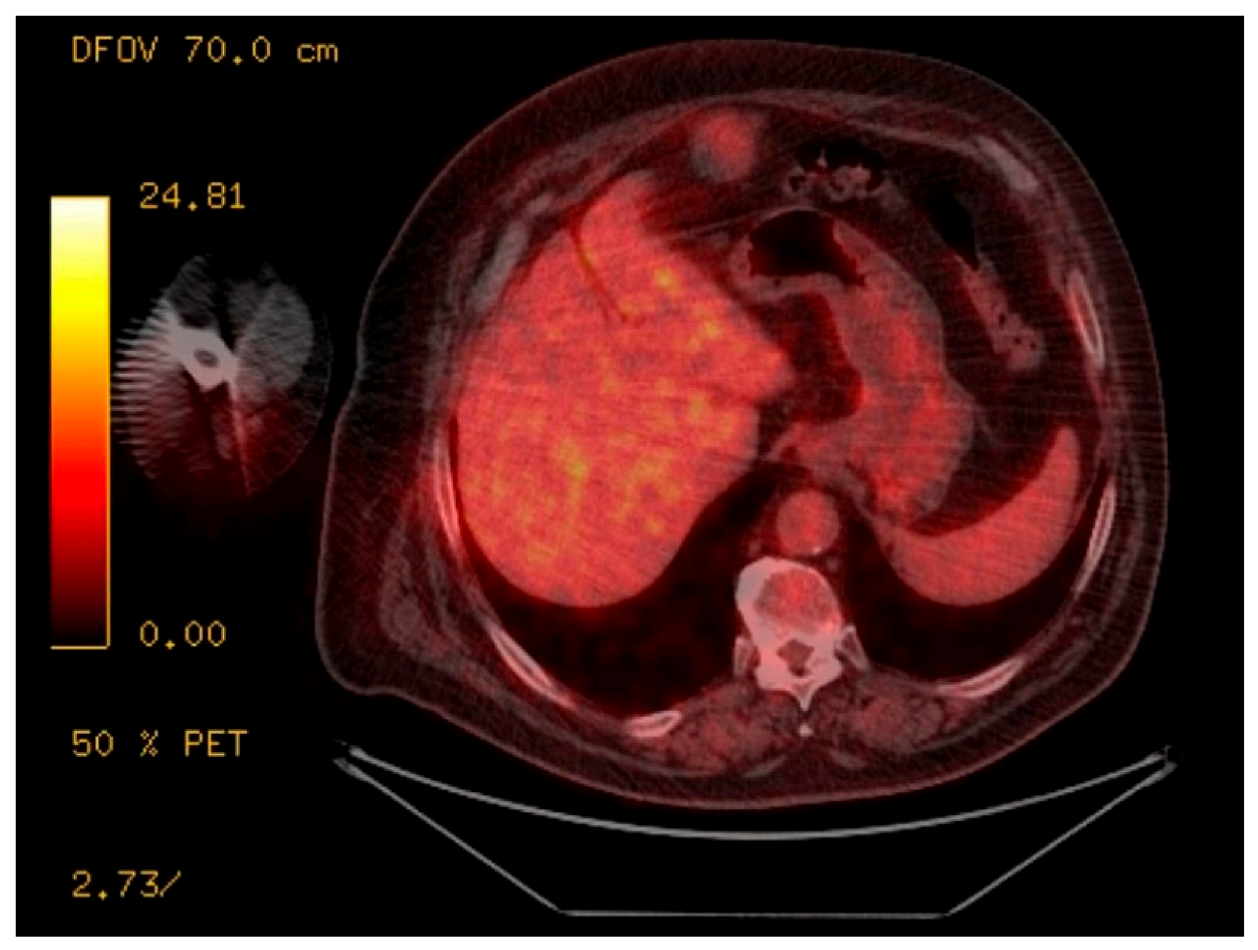
Preoperative axial fused PET/CT caption through the upper abdomen, inferior to the principal cardiac structures.

**Figure 9:**
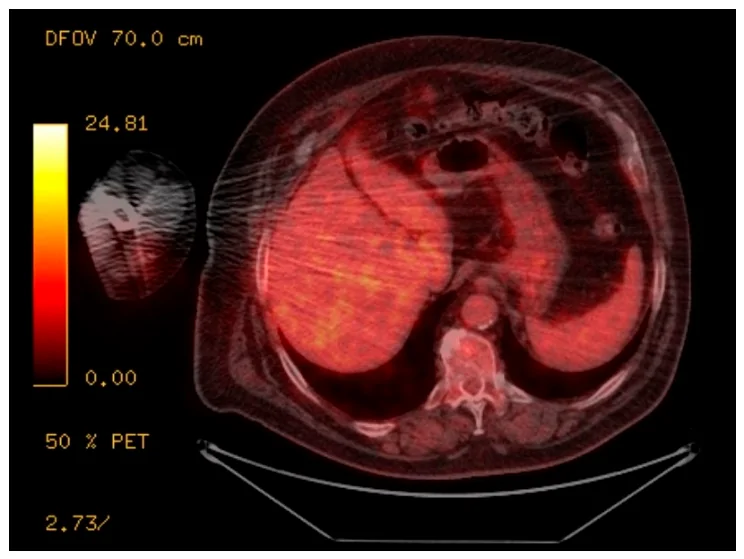
Preoperative axial fused PET/CT image at the level of the upper abdomen.

**Figure 10:**
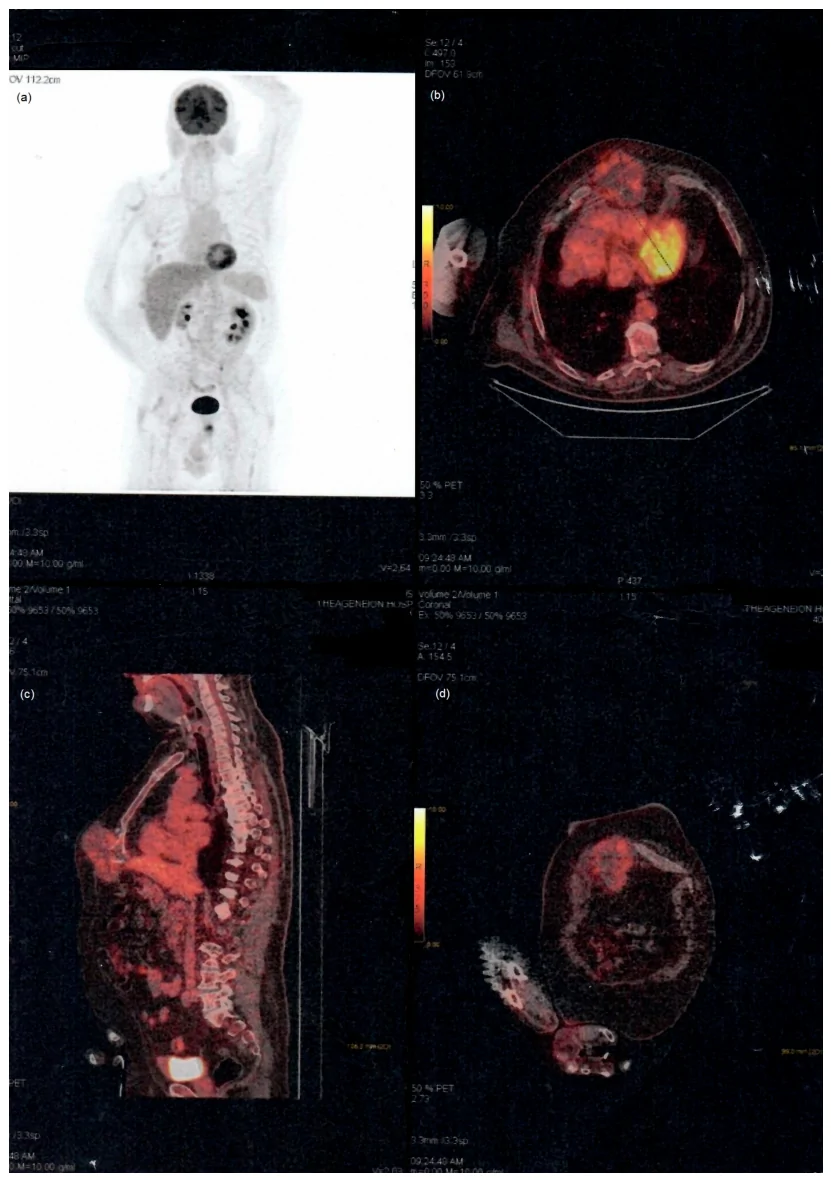
Multiplanar preoperative PET/CT imaging, including (**a**) Whole-body maximum-intensity projection PET caption in inverted grayscale, (**b**) Axial fused PET/CT caption through the lower thorax, (**c**) Sagittal fused PET/CT reconstruction and (**d**) Axial fused PET/CT caption at the level of the lower anterior chest wall/xiphoid region.

The abovementioned symptomatology was described as gradual painful swelling at the level of the sternum. Because of the anterior location of the lesion and the progressive nature of the symptoms, diagnostic imaging was initiated. The patient underwent contrast-enhanced CT (Figure 2) and PET–CT Control (Figure 3, Figure 4, Figure 5, Figure 6, Figure 7, Figure 8, Figure 9 and Figure 10).

These examinations revealed a large heterogeneous mass located at the anatomical border between the anterior thoracic wall and the upper abdominal wall. The lesion involved the lower sternal region and infiltrated the xiphoid process. Its size, heterogeneity, anatomical location, and infiltrative features raised strong suspicion of a primary malignant chest wall tumor. The tumor presented significant measurements (DMax: 10.1 cm) with a mediocre Standardized Uptake Value (SUVMax: 4.9).

The use of multiple imaging modalities was crucial for surgical planning. CT provided information regarding the osseous extent, bony involvement and the anatomical relationship of the mass to the sternum, costal arches, diaphragm and anterior abdominal wall. PET-CT was performed as part of systemic staging, metabolic assessment and biopsy targeting. No preoperative MRI control, three-dimensional segmentation, printed model, or custom implant planning was performed due to the unavailability at our institution at the time. Given the uncommon location and the need for tissue confirmation, a CT-guided (FNA) biopsy was performed successfully. Histopathological examination identified the mass as grade III chondrosarcoma.

The diagnosis of high-grade chondrosarcoma established the need for radical surgical treatment. In view of the size and position of the lesion, its infiltration of the xiphoid process, and its extension across the anterior thoracoabdominal junction, a minimally invasive approach was not considered appropriate for oncologically adequate resection. The case was evaluated with the principles of multidisciplinary oncologic care, involving thoracic surgeons, radiologists, pathologists, anesthesiologists, intensivists, and oncologists. The absence of a plastic/reconstructive surgeon’s participation is due to the unavailability of an in-house plastic surgery physician’s service at our regional referral hospital. Eventual transfer for planned flap reconstruction and procurement of a custom or hybrid rigid implant were considered impractical because they would have delayed definitive oncologic surgery (Table 1). The operative strategy was based on the need to achieve extensive exposure, remove the tumor “en bloc” with involved skeletal structures, and reconstruct the resulting chest wall defect.

**Figure 11:**
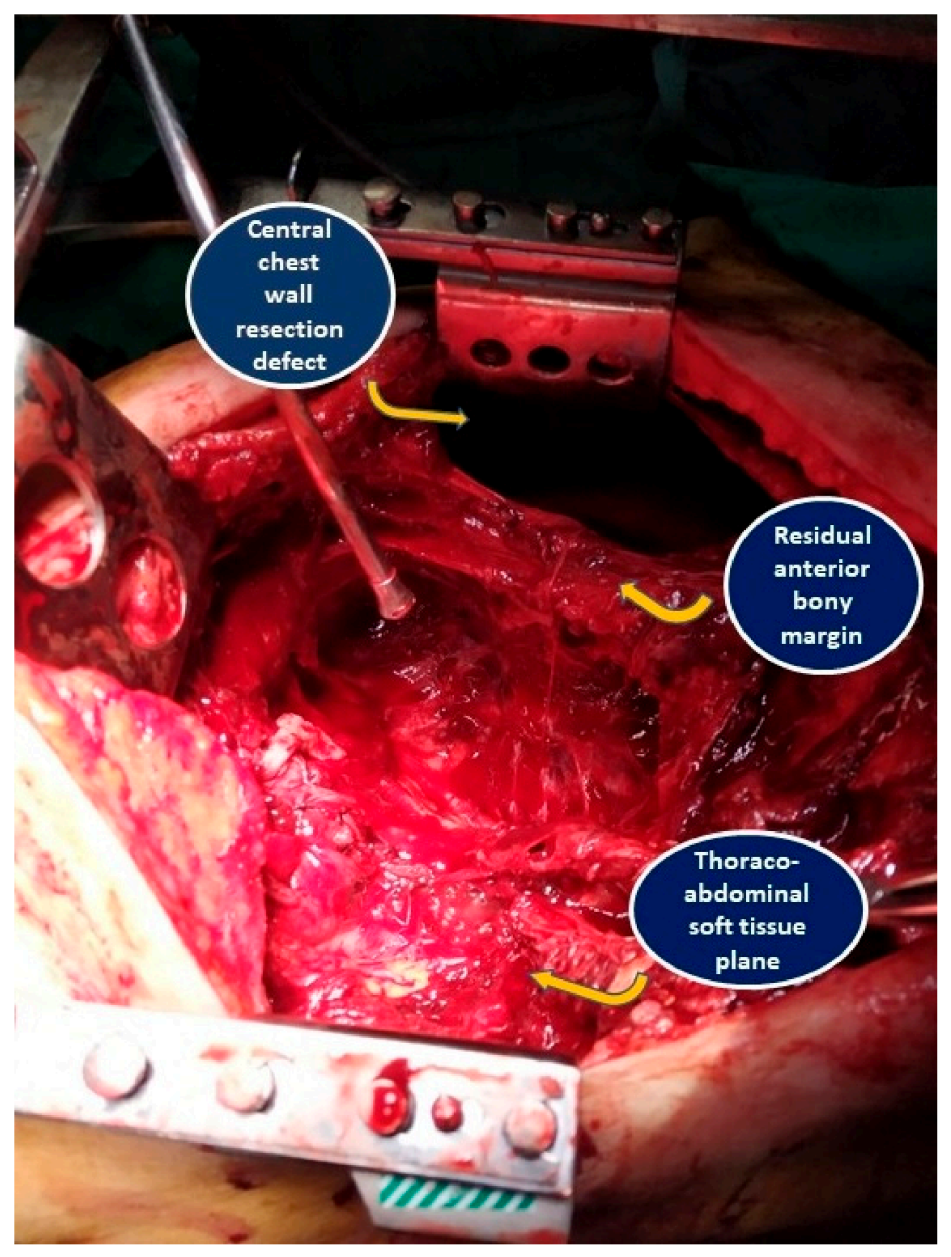
Intraoperative view after “en bloc” resection, showing the central chest wall defect, residual anterior bony margin, and thoracoabdominal soft-tissue plane.

**Figure 12:**
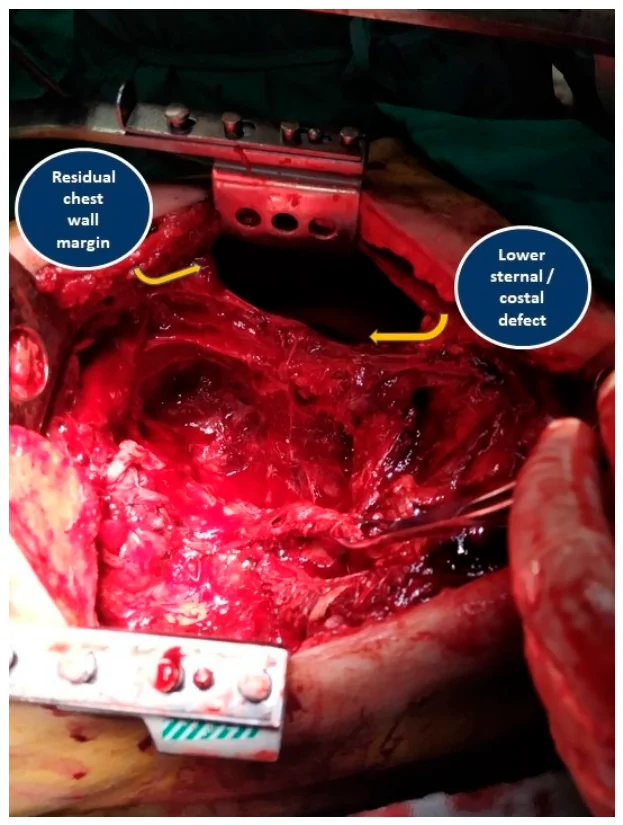
Intraoperative view of the lower sternal and costal-arch resection defect.

The patient underwent bilateral anterior thoracotomy through the “Clamshell” approach, AKA bilateral transverse thoraco-sternotomy, at the level selected from preoperative imaging. After placement in supine position, the procedure provided broad exposure of the anterior thoracic cavity and lower sternum, allowing controlled access to both hemithoraces and the central chest wall. The tumor was resected “en bloc” together with the middle and lower portions of the sternum and adjacent costal-arch segments (Figure 11 and Figure 12). The operation included removal of the involved xiphoid process and surrounding soft tissues judged to be necessary for macroscopic oncologic clearance. The upper sternum and lateral bony arches were preserved. The resulting defect required immediate reconstruction.

**Figure 13:**
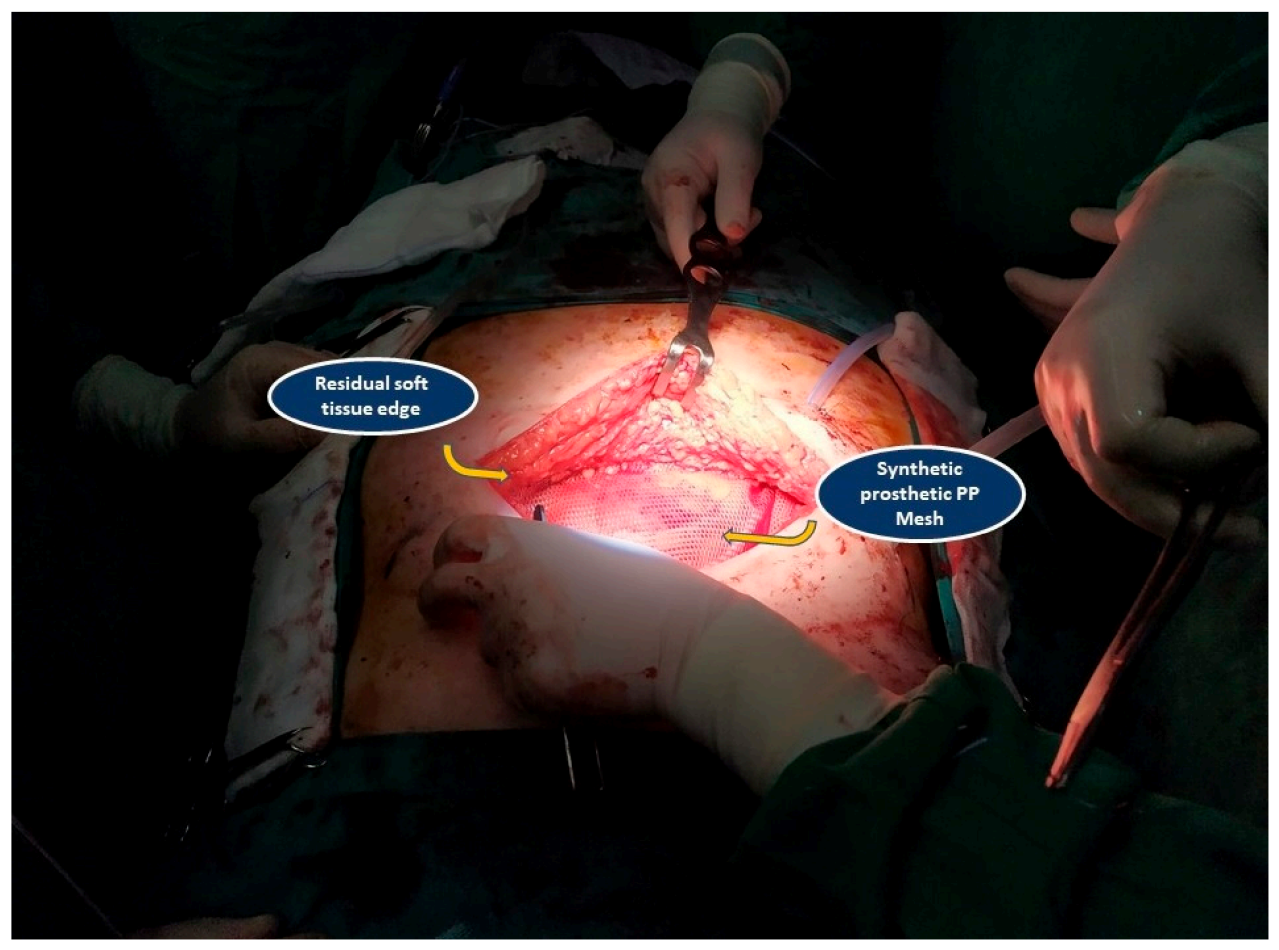
Intraoperative view of the synthetic PP prosthetic mesh.

**Figure 14:**
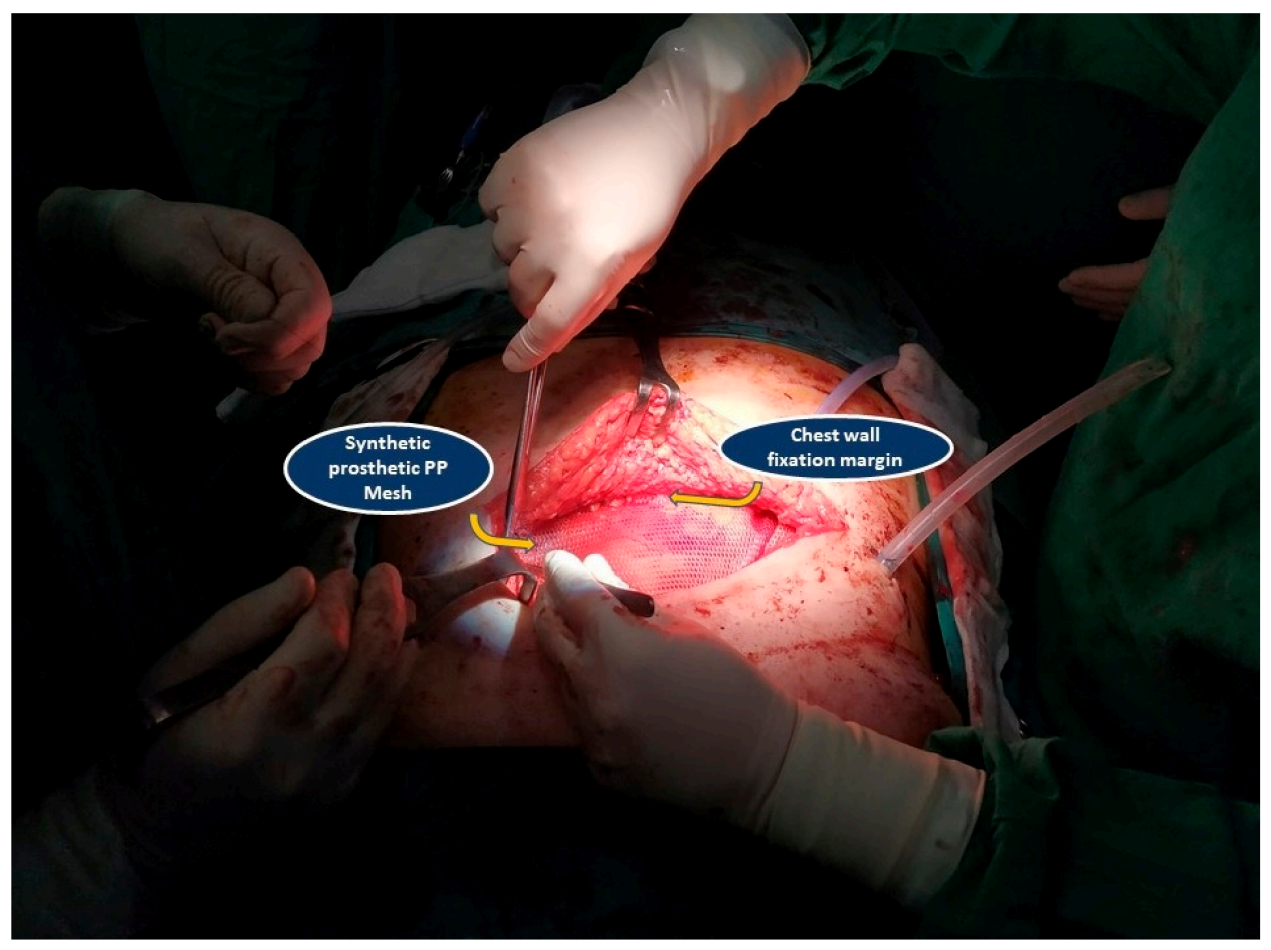
Intraoperative view of the synthetic PP prosthetic mesh and its circumferential fixation to the residual chest wall margins.

A synthetic polypropylene (PP) prosthetic mesh was tailored to bridge the lower anterior defect and was fixed circumferentially to the remaining osseous and musculoaponeurotic margins, in order to support respiratory mechanics and protect the intrathoracic structures (Figure 13 and Figure 14). The overall procedure lasted approximately 6.5 h (~400 min) of net surgical time, with no significant intraoperative manifestations of respiratory disorder. The patient’s intraoperative needs for blood transfusion did not exceed the provision of two blood units.

**Figure 15:**
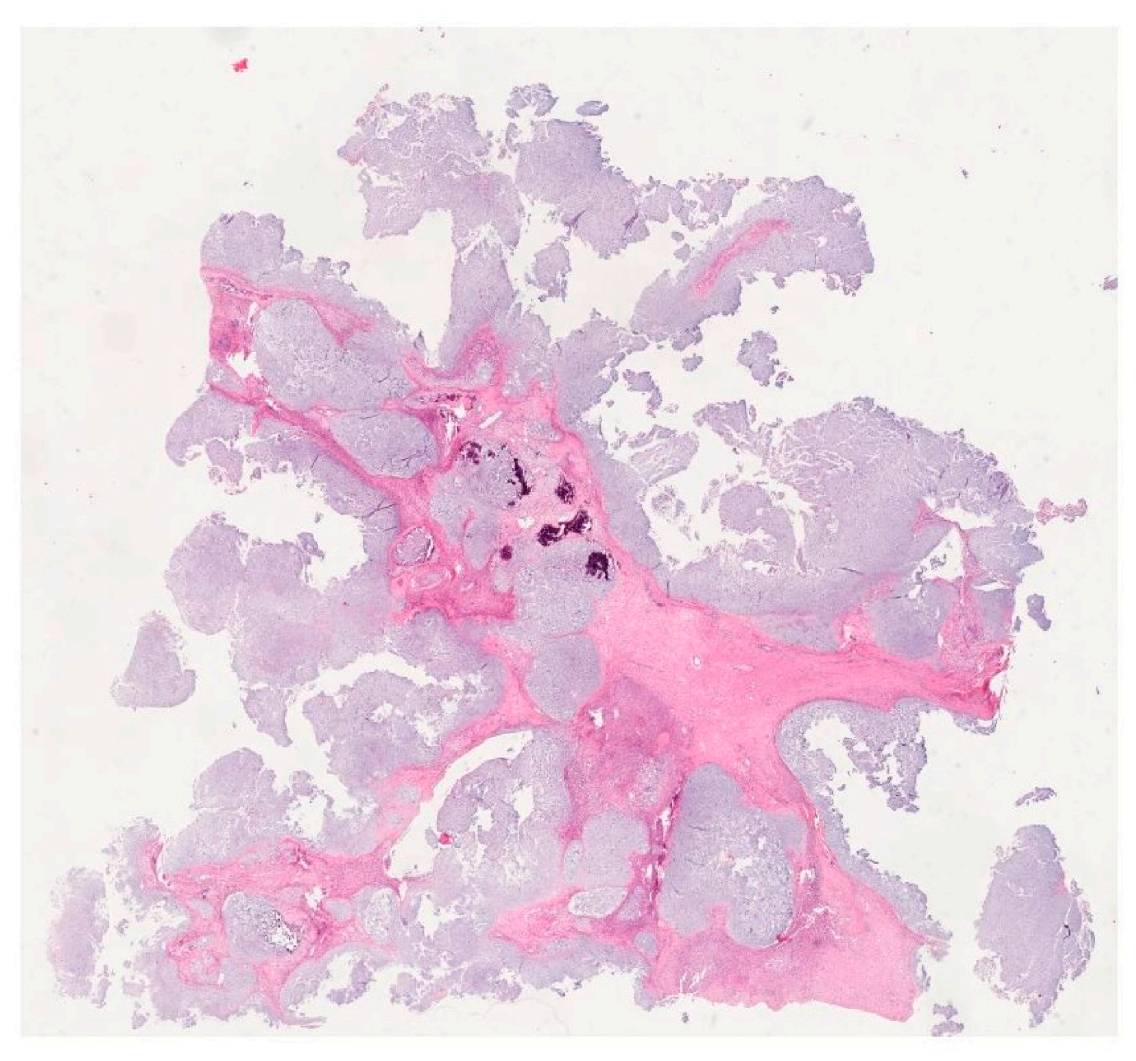
Histological caption for the extracted grade III chondrosarcoma tissue.

**Figure 16:**
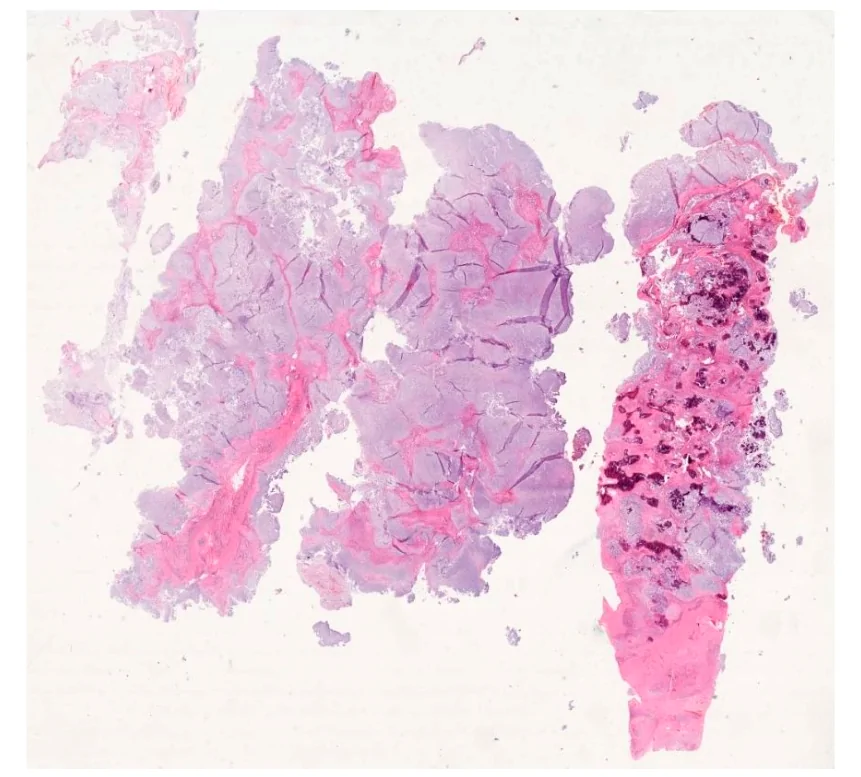
Histological caption for the extracted grade III chondrosarcoma tissue.

The following histological study confirmed the lesion’s already predefined type, a grade III chondrosarcoma. The resected specimen measured approximately 9 cm at gross pathological assessment, compared with 10.1 cm on preoperative imaging (Figure 15 and Figure 16). No tumor was present at the examined margins, corresponding to an R_0_ resection. The closest reported microscopic clearance was approximately 10 mm. The pathology report did not provide separate numerical distances for the pleural or peritoneal, or each individual bony resection plane.

Postoperatively, the patient was transferred to the intensive care unit (ICU) for postoperative monitoring and hemodynamic and ventilatory support. The ICU stay lasted 18 days. During this period, his recovery was complicated by failure to achieve complete awakening and intermittent oxygen desaturation episodes. Because of prolonged ventilatory dependence and the need to secure the airway, a tracheostomy was performed on the 8th postoperative day. The respiratory course was considered multifactorial, reflecting the magnitude of the bilateral anterior thoracotomy, loss of lower anterior chest wall support, severe obesity, smoking-related vulnerability, and prolonged immobilization.

After stabilization in the ICU, the patient was transferred back to the thoracic surgery ward. The postoperative course was subsequently complicated by wound dehiscence, which led to surgical revision of the disrupted postoperative wound on the 29th postoperative day. A second revision was performed on the 44th postoperative day because of persistent wound-related problems. During the second revision, the prosthetic mesh was removed due to persistent febrile episodes and a positive wound culture for Acinetobacter Baumanii and Staphylococcus Epidermidis. The need to remove the mesh illustrates one of the central dilemmas in extensive chest wall surgery. Prosthetic reconstruction is often essential for restoring thoracic stability after large anterior resections. However, prosthetic material can become a focus of infection, particularly in patients with wound dehiscence, prolonged hospitalization, respiratory compromise, obesity, or repeated surgical interventions. In such circumstances, removal of the prosthetic material becomes necessary, despite the reconstructive disadvantage, because control of infection is a prerequisite for recovery. After mesh removal, infection control and soft-tissue healing took priority over immediate reimplantation. No acute paradoxical motion or visceral herniation requiring emergency rigid reconstruction was documented during the remaining admission.

**Figure 17:**
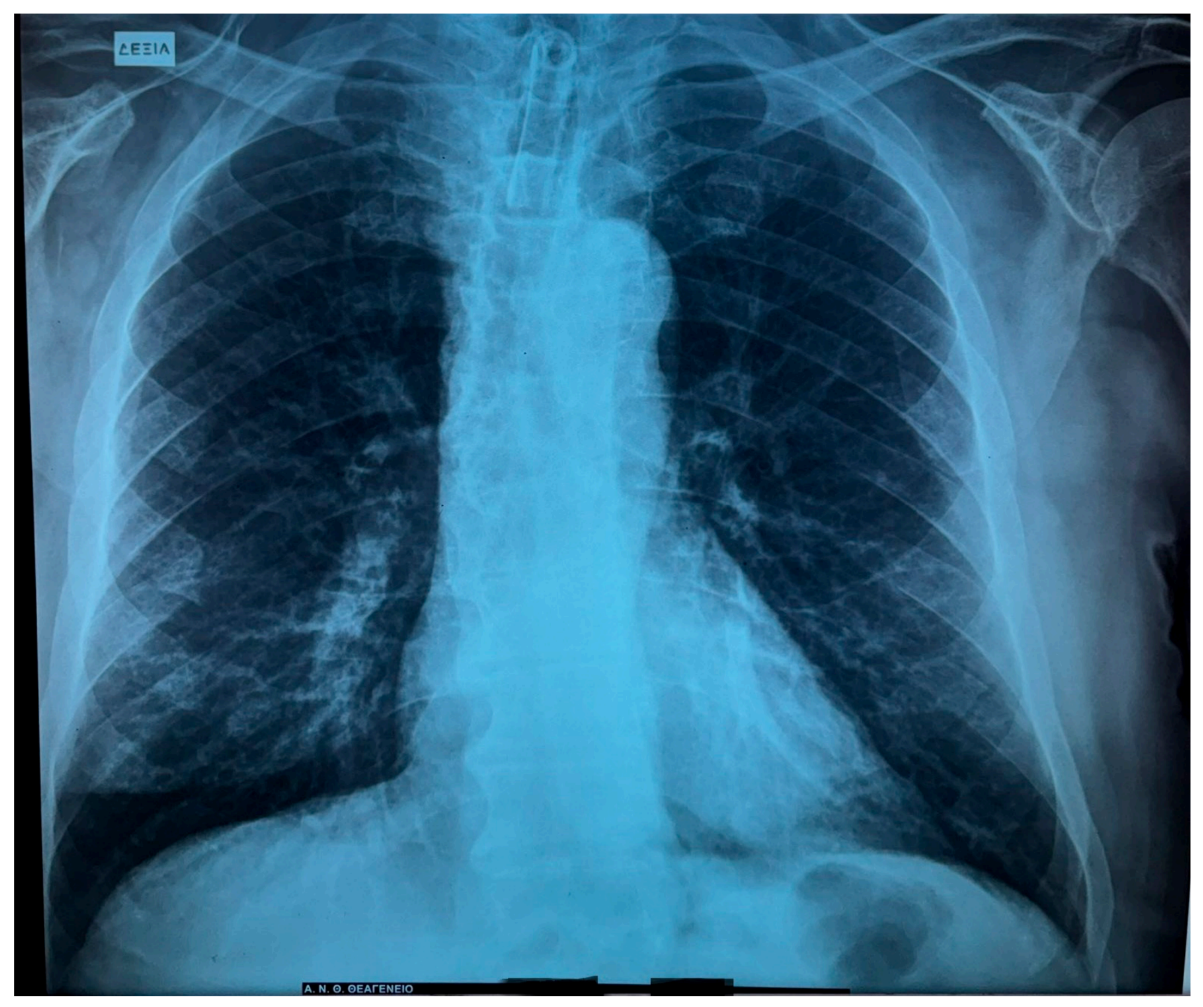
The 64th postoperative day X-Ray control before tracheostomy removal.

The tracheostomy was removed on the 64th postoperative day (Figure 17).

The patient was discharged two days later in a stable condition, following prolonged hospitalization and gradual clinical improvement. Since discharge, he remains under regular thoracic surgical and oncologic surveillance, and is currently in his third postoperative year. No clinical signs of recurrence are recorded to this day, either surgical or oncological. The patient’s follow-up is being conducted under high-grade bone-sarcoma principles, with examination and local/chest imaging every 3 months for 2 years, every 6 months during years three to five, and annually thereafter [10]. Ongoing surveillance is essential because high-grade chondrosarcoma of the chest wall carries a significant risk of local recurrence and distant spread, and because postoperative functional recovery after extensive chest wall resection may continue for a prolonged period.

## 3. Discussion

This case represents a rare and surgically demanding presentation of high-grade chondrosarcoma involving the lower sternum and xiphoid process [11]. Tumors in this area may extend across conventional anatomical boundaries and create combined thoracic and abdominal reconstructive challenges. Unlike more lateral rib tumors, which can often be managed through localized chest wall resection, tumors of the lower sternum require bilateral anterior exposure, central skeletal resection, and immediate reconstruction [12]. Regarding chest wall chondrosarcomas, negative margins remain a central oncologic objective. In a national cohort, positive margins were associated with worse survival (hazard ratio, 2.34), and a 2026 multicenter study of 147 patients found that a microscopic clearance of at least 4 mm significantly reduced local recurrence (hazard ratio, 0.12) [13]. Our patient’s specimen indicated an approximately 10 mm closest reported clearance and no tumor at the examined margins (R_0_), although plane-specific measurements were unavailable. Having in mind the rarity of such chondrosarcomas, several principles still remain consistent [14]. First, chest wall chondrosarcomas should be treated with curative intent whenever complete resection is feasible [15]. Second, inadequate margins increase the risk of local recurrence. Third, the reconstructive strategy must be individualized according to the size and location of the defect, the number of ribs or costal cartilages removed, sternal involvement, respiratory mechanics, infection risk, and available soft-tissue coverage [16].

The surgical approach is one of the most instructive aspects of this case. Minimally invasive thoracic surgery (MITS) techniques are now considered the “state of the art” and a major advance, but they should not be viewed as a universal substitute for open surgery. The optimal surgical method is the one that allows safe, complete, and anatomically appropriate treatment of the disease. For a large anterior chest wall sarcoma infiltrating the lower sternum and xiphoid process, a limited approach could compromise exposure, margin control, and reconstruction. A grade III diagnosis justifies an aggressive surgical strategy, provided that the patient can tolerate the procedure and that the anticipated resection is technically feasible [17]. In this setting, the “Clamshell” procedure was selected because it offered extensive bilateral access and allowed “en bloc” resection of the tumor with involved skeletal structures, such as the lower sternum, adjacent costal arches, and anterior thoracoabdominal junction in a controlled manner in this case. The patient’s obesity and heavy smoking history increased the perioperative risk, but these factors did not negate the oncologic need for radical management. The postoperative complications in this case were significant but understandable in view of the patient’s risk profile and the extent of surgery. The abovementioned highlight an important principle. Older or less frequently used surgical approaches remain valuable when dictated by anatomy and oncologic necessity [18].

Reconstructive methods in such cases must separately address skeletal stability and vascularized soft-tissue coverage. Such reconstruction after oncologic resection must aim for the protection of intrathoracic organs, preservation of respiratory mechanics, prevention of paradoxical movement, reduction in the risk of herniation, and provision of a stable platform for soft-tissue healing. In lower sternal and xiphoid resections, the balance between rigidity and flexibility is particularly important, because excessive rigidity may impair mechanics while inadequate support may compromise stability [19]. Flexible synthetic meshes are adaptable, radiolucent, and relatively inexpensive but provide less resistance to paradoxical movement than titanium plates, titanium mesh, methyl-methacrylate sandwiches, or patient-specific implants. Hybrid rigid constructs are generally attractive for large central/anterior defects, whereas mesh only or semirigid repair can be adequate in selected partial sternectomies. In a 14-patient sternal-tumor series with an average defect of 75 cm^2^, most reconstructions were semirigid- or suture-based and only one used rigid methyl methacrylate. The outcomes were acceptable, although one patient required prolonged ventilation [20]. Additionally, full thickness chest wall reconstruction indicates substantial morbidity. A 42-case observational study reported complications in 52.4%, including wound complications in 21.4%, pulmonary complications in 19.0%, and reoperation in 11.9% [21]. These data support individualized selection rather than a single mandatory material. For this patient, a rigid plate mesh construct was considered but not used. The familiar prosthetic synthetic PP mesh was immediately available and applied, not as a compromise, since the preserved upper sternum and lateral costal arches provided residual support, while the defect was predominantly caudal and could be bridged with mesh.

Wound dehiscence and prosthetic infection are among the most challenging complications after chest wall reconstruction, especially in oncologic patients. Such complications may also delay adjuvant treatment when indicated and prolong hospitalization [22]. Therefore, preoperative planning should include not only the resection and reconstruction itself, but also anticipation of possible failure scenarios. In high-risk patients, early involvement of plastic or reconstructive surgeons proves beneficial, especially when large defects, limited soft-tissue coverage, or prosthetic reconstruction are expected. Once infection involves the prosthesis, eradication and soft tissue healing appropriately takes precedence over immediate reimplantation. Delayed reconstruction should be symptom-driven after infection control and should use rigid support and vascularized tissue if instability, pain, paradoxical motion, or herniation become clinically important. Nevertheless, the use of custom implants or/and hybrid titanium systems is clearly indicative in modern thoracic/chest wall surgery. A 26-patient titanium-mesh series reported a mean operative time of 315 min, 19% overall morbidity, and 4% reconstructive failure, illustrating both the potential value and the resource intensity of rigid systems [23]. In this case, the abovementioned absence of reconstructive/plastic input presented important limitations that may have reduced options for vascularized coverage, although causation cannot be isolated from severe obesity, smoking, prolonged ventilation, repeated procedures, and prosthetic contamination. Three-dimensional segmentation or printing clarifies resection geometry and enables pre-contoured or custom reconstruction, while prospective thoracic–oncoplastic strategies associate coordinated flap planning with low surgical site infection rates, particularly when rigid material is implanted [24].

The diagnostic pathway also deserves emphasis. This patient initially presented with a gradually painful swelling, a symptom that may appear nonspecific but should not be underestimated when located over the sternum or anterior chest wall. A palpable, painful, enlarging chest wall mass in an adult requires thorough investigation. In this context, CT, MRI, and PET-CT imaging control offer complementary information. The contribution of the radiologists, interventional or not, extends beyond diagnosis and influences biopsy planning, operative strategy, and postoperative surveillance [25]. CT-guided biopsy is often preferred when it can safely access representative tumor tissue. In heterogeneous lesions, targeting the most aggressive-appearing region may improve the diagnostic yield. The present case demonstrates the value of image-guided tissue acquisition in establishing the diagnosis before a major operation. Biopsy must be planned so that the tract can be excised during definitive surgery whenever possible. Poorly placed biopsies may contaminate tissue planes or complicate subsequent resection [26]. Pathologists are equally central to management. Chondrosarcoma diagnosis requires tasks such as recognition of malignant cartilage production, assessment of cellularity, nuclear atypia, mitotic activity, permeative growth, necrosis, and grade, particularly in limited biopsy material. In the present case, CT-guided biopsy was sufficient to identify grade III disease and direct the team toward radical surgery [27]. Oncologists contribute to staging, evaluation of histological subtype, consideration of systemic therapy in selected variants, assessment of metastatic risk, and surveillance planning. The biological behavior of chondrosarcoma differs substantially from other sarcomas and is generally less responsive to cytotoxic chemotherapy and radiotherapy. This relative resistance has a direct impact on clinical decision making. Surveillance should evaluate both oncologic and functional outcomes. Oncologic follow-up includes imaging for local recurrence, pulmonary metastases, and distant disease according to relevant protocols and individualized risk [28]. Functional follow-up assesses respiratory status, chest wall stability, wound healing, pain, shoulder and thoracoabdominal mobility, nutritional recovery, and quality of life. In patients who undergo mesh removal, careful clinical and radiologic monitoring is needed to identify late instability, herniation, or functional impairment.

The value of this case lies in its complexity. It combines a rare anatomical site, high-grade sarcoma biology, major open thoracic access, chest wall reconstruction, prolonged intensive care, airway management, wound complications, prosthetic infection, revision surgery, decannulation, and continued multidisciplinary follow-up [29]. Such cases remind clinicians that the successful treatment of rare thoracic malignancies is rarely the result of a single intervention. It is the cumulative outcome of accurate diagnosis, appropriate surgical judgment, perioperative resilience, complication management, and sustained oncologic surveillance. Multidisciplinary teams (MDT) must include thoracic surgeons, musculoskeletal oncologic surgeons when available, radiologists, pathologists, medical oncologists, radiation oncologists, anesthesiologists, intensivists, infectious disease specialists, rehabilitation physicians, and reconstructive surgeons [30].

## 4. Conclusions

High-grade chondrosarcoma involving the lower sternum and xiphoid process is an uncommon and challenging entity. In such rare cases, individualized treatment, multidisciplinary collaboration, and readiness to combine traditional surgical exposure with modern oncologic principles remain decisive for patient care. Surgery remains the principal curative modality, particularly when the disease is localized and technically resectable, but the postoperative course also emphasizes that radical surgery does not end in the operating room, requiring coordinated participation from every contributing medical specialty field.

This case demonstrates that extensive open surgical approaches continue to have an essential role in modern thoracic oncology, when MITS procedures have evolved as the preferred approach for the majority of malignant conditions. The “Clamshell” bilateral thoracotomy provided the access required for “en bloc” R_0_ resection of a complex anterior thoraco-abdominal chest wall tumor, enabling immediate reconstruction, which must always be matched to defect mechanics, infection risk and soft-tissue coverage. In this report, mesh-only repair achieved immediate closure but was followed by dehiscence and infection requiring wound revision and mesh explantation. The experience supports early planning with chest wall and plastic/reconstructive specialists when available, transparent reporting of resource limitations, and long-term surveillance for recurrence and delayed mechanical failure.

## Figures and Tables

**Table 1:** MDTs’ participant specialties and their overall contribution.

Specialty	Contribution
Internal Medicine	Initial workup and referral
Oncology	Risk stratification and treatment planning
Radiology	Imaging and guided biopsy
Pathology	Histology, Immunohistochemistry and molecular testing
Infectious Diseases	Evaluation of granulomatous/infectious etiologies
Thoracic Surgery	Surgical access, staging, and resection
Plastic/Reconstructive Surgery	No in-house service; therefore, no involvement in the index operation. External referral was preferred if delayed rigid/flap reconstruction becomes necessary.

## Data Availability

The original contributions presented in this study are included in the article. Further inquiries can be directed to the corresponding author.

## Abbreviations

BMI	Body Mass Index
CT	Computed Tomography
DMax	Maximum Diameter
FNA	Fine-Needle Aspiration
ICU	Intensive Care Unit
MDT	Multidisciplinary tumor board
MITS	Minimally Invasive Thoracic Surgery
MRI	Magnetic Resonance Imaging
PET-CT	Positron Emission Tomography–Computed Tomography
PP	Polypropylene
RATS	Robotic-Assisted Thoracic Surgery
SUV	Standardized Uptake Value
VATS	Video-Assisted Thoracic Surgery
